# Blood Pressure, Urinalysis, and Edema Assessment in Perinatal Care: From Historical Foundations to Evidence-Based Practice

**DOI:** 10.31662/jmaj.2025-0241

**Published:** 2025-11-21

**Authors:** Yoshitsugu Chigusa, Kazuki Yamano, Taito Miyamoto, Haruta Mogami, Masaki Mandai, Hirohito Metoki, Akihiko Sekizawa

**Affiliations:** 1 Department of Gynecology and Obstetrics, Graduate School of Medicine, Kyoto University, Kyoto, Japan; 2 Division of Obstetrics, Center for Maternal-Fetal, Neonatal and Reproductive Medicine, National Center for Child Health and Development, Tokyo, Japan; 3 Division of Public Health, Hygiene and Epidemiology, Faculty of Medicine, Tohoku Medical and Pharmaceutical University, Sendai, Japan; 4 Department of Obstetrics and Gynecology, Showa Medical University School of Medicine, Tokyo, Japan

**Keywords:** blood pressure, edema, Maternal and Child Health Handbook, perinatal care, urinalysis

## Abstract

The triad of blood pressure measurement, urinalysis, and edema assessment has constituted the cornerstone of prenatal care for over eight decades and has been consistently recorded at each examination in Japan’s Maternal and Child Health Handbook. Historically, these examinations were prioritized to facilitate the early diagnosis and treatment of pregnancy toxemia―now termed hypertensive disorders of pregnancy―which once represented a leading cause of maternal mortality. The triad has undeniably played a pivotal role in the early diagnosis, prediction, and treatment of hypertensive disorders of pregnancy, while potentially contributing to the detection of other obstetric complications. However, conclusive evidence linking these conventionally performed assessments to actual pregnancy and delivery outcomes remains limited, and critical unresolved issues persist within each examination category. Blood pressure assessment requires the establishment of optimal blood pressure thresholds to achieve favorable pregnancy and perinatal outcomes; to achieve this, home blood pressure monitoring should be adopted. Evidence-based target blood pressure ranges for treatment initiation in hypertensive disorders of pregnancy and therapeutic targets are urgently needed. For urinary protein assessment, the establishment of standardized criteria for postpartum proteinuria follow-up is essential. In terms of edema, critical evidence is lacking regarding the evaluation and treatment of pathological edema that adversely affects pregnancy outcomes. This review traces the historical evolution of these examinations while critically examining their contemporary role in perinatal care. A notable characteristic of this triad is its noninvasive nature and minimal cost burden. The establishment of robust evidence supporting their clinical utility could optimize maternal and fetal outcomes while maintaining cost-effective healthcare delivery.

## Introduction

**Figure 1.**
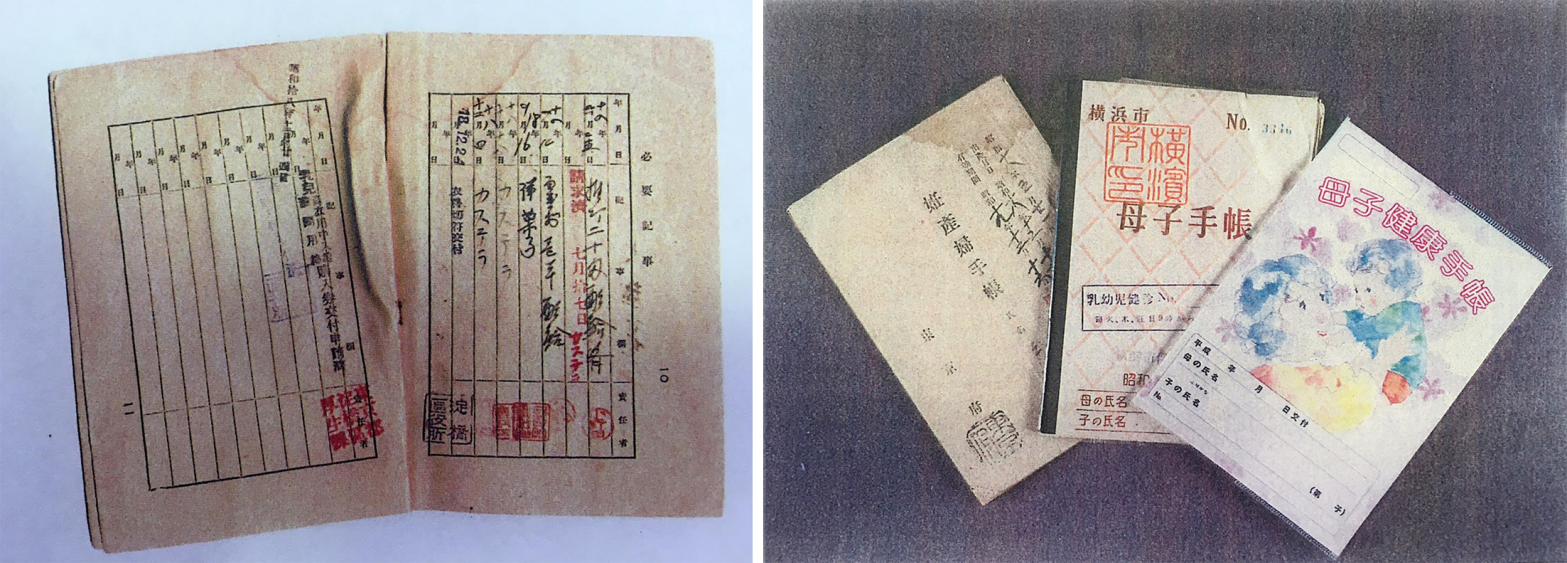
Informational leaflet for pregnant and postpartum women and the Maternal and Child Health Handbooks. Due to material shortages during World War II, pregnant and postpartum women could receive priority distribution of supplies by presenting this leaflet (left). The document contains records showing the distribution of castella cakes and the issuance of clothing ration tickets (left). After the war, this leaflet was renamed the “Maternal and Child Health Handbook” and revised to include documentation of medical examinations, laboratory findings during pregnancy, and health guidance information (right). This continues to be issued to pregnant women to this day.

In Japan, the Maternal and Child Health Act mandates the issuance of a Maternal and Child Health Handbook to women upon notification of pregnancy. This handbook originated as an informational leaflet for pregnant and postpartum women and was first distributed in 1942 during World War II (Figure 1). It has remained an essential resource for women undergoing prenatal examinations for over eight decades (Figure 1). Although the structure, content, and examinations of prenatal care have undergone significant transformations during this period, the “Pregnancy Progress” section of the handbook still contains three fundamental assessment parameters that must be recorded at each examination: blood pressure (BP), urinalysis (glucose and protein), and edema. Thus, these three parameters are considered cornerstone assessments of prenatal examinations in Japan.

According to the “Guidelines for Maternal Health,” published in 1950, during the early implementation of the Maternal and Child Health Handbook, the three primary causes of maternal mortality were puerperal fever, obstetric hemorrhage, and pregnancy toxemia, now termed hypertensive disorders of pregnancy (HDP). These three conditions collectively accounted for more than 60% of maternal deaths in Japan at the time. Furthermore, analysis of the causes of stillbirth revealed that pregnancy toxemia was implicated in 11% of cases, representing the most prevalent etiological factor. In response to these concerning statistics, the Maternal and Child Health Policy Framework issued in 1948 explicitly prioritized “efforts toward prevention, early diagnosis, and prompt treatment of pregnancy toxemia to reduce maternal and fetal mortality.”

The establishment of BP, urinalysis, and edema assessment as fundamental components of prenatal examinations was largely influenced by this historical context. However, as medical science has advanced, pregnancy toxemia has been reclassified as HDP. Although not yet fully understood, significant progress has been made in clarifying its etiology, predicting its onset, and developing effective treatment modalities.

The purpose of this review is to pay due respect to the pivotal role these three examinations have played in the historical campaign against pregnancy toxemia, while critically reassessing whether they maintain their clinical significance in contemporary obstetric practice. While acknowledging their historical importance, this study seeks to question and evaluate whether these traditional parameters remain relevant amidst the evolution of modern fetal and maternal medicine.

## Blood Pressure

### Overview of BP assessment

#### BP classification and measurement techniques

**Figure 2.**
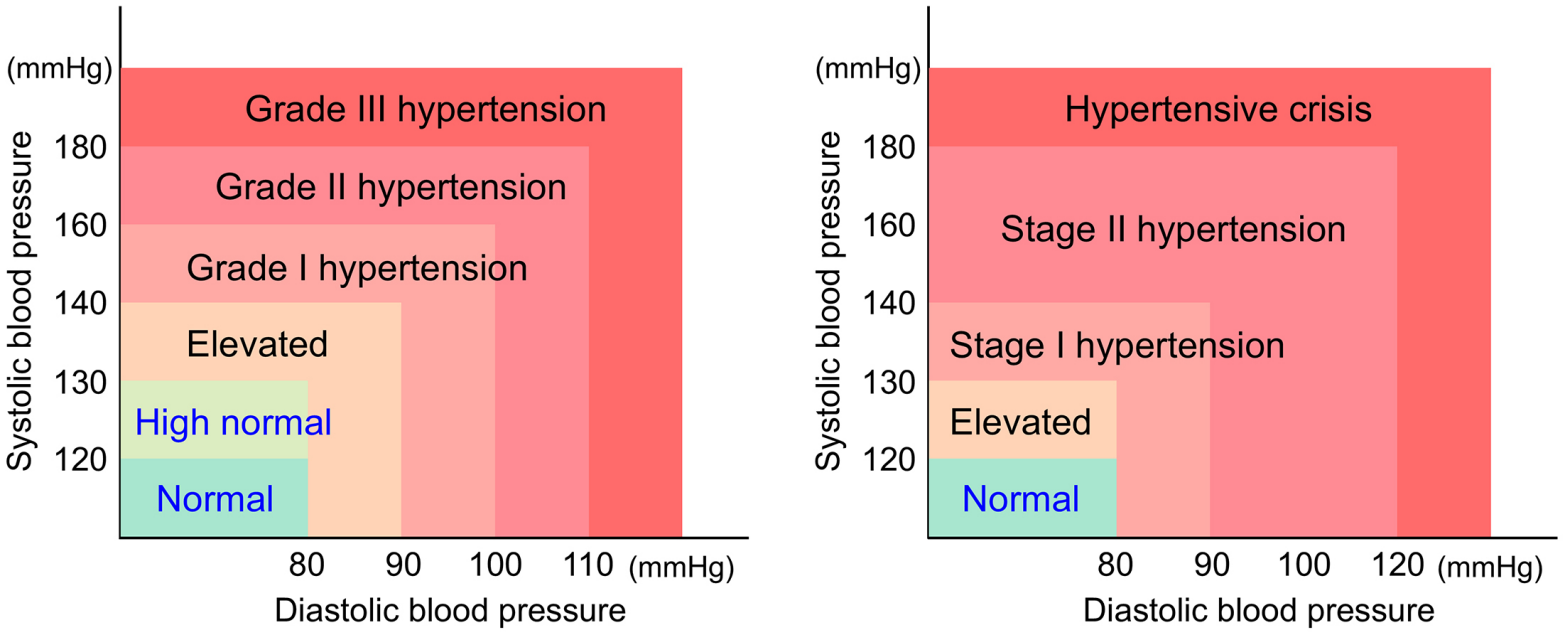
Difference in blood pressure categorization between Japan and the United States. Blood pressure categories based on the guidelines for the management of hypertension published by the Japanese Society of Hypertension (left) and the guidelines for the prevention, detection, evaluation, and management of high blood pressure in adults published by the American College of Cardiology and American Heart Association (right).

Figure 2 illustrates the classification of BP levels in Japan ^(1)^ and those defined by the American College of Cardiology and American Heart Association (AHA) ^(2)^. In Japan, hypertension is classified as a systolic BP (sBP) ≥140 mmHg and/or a diastolic BP (dBP) ≥90 mmHg ^(1)^. However, the AHA defines hypertension as an sBP ≥130 mmHg or a dBP ≥80 mmHg ^(2)^, which represents a lower threshold compared to the Japanese classification. During pregnancy, an sBP ≥140 mmHg or a dBP ≥90 mmHg is considered pathological and is diagnosed as HDP ^(3)^. In particular, an sBP ≥160 mmHg and/or a dBP ≥110 mmHg is classified as a severe feature of HDP ^(3)^.

In clinical practice, BP is commonly measured using either the auscultatory method with an electronic sphygmomanometer or a spring-based aneroid sphygmomanometer or by means of an automatic upper-arm BP monitor. The protocol for BP measurement during pregnancy, particularly for the diagnosis of HDP, is outlined in Table 1.

**Table 1.** Blood Pressure Measurement Protocol.

Measurement device	Use an electronic column (pseudo-mercury) sphygmomanometer, an aneroid sphygmomanometer with auscultation method, or an automated upper arm blood pressure monitor.
Pre-measurement restrictions	Avoid caffeine intake and smoking within 30 minutes before measurement.
Measurement position	Sit on a chair with back support without crossing legs, and rest for at least 5 minutes before measurement.
Cuff position	Ensure the cuff wrapped around the arm is at heart level.
Measurement frequency	Take two measurements in a seated position at 1-2 minute intervals and use the average value.
Measurement variability	If the second measurement differs by ≥5 mmHg, continue measuring until stabilized.
Bilateral measurement	At initial assessment, measure blood pressure in both arms; if the difference is ≥10 mmHg, adopt the higher value.

#### BP variability during pregnancy and the postpartum period

During pregnancy, physiological changes lead to a decrease in peripheral vascular resistance, primarily due to the vasodilatory effects of hormones such as progesterone and relaxin. As a result, BP decreases from early to mid-pregnancy ^(4)^. Subsequently, BP increases slightly because of the rise in circulating plasma volume; however, in normal pregnancies, BP remains within the normal range and does not exhibit a significant increase ^(5)^. A multicenter collaborative study conducted in Japan involving 218 participants confirmed that BP decreased from early to mid-pregnancy, reached its lowest level at 17-21 weeks of gestation, increased after 24 weeks, and returned to nonpregnant levels by 4 weeks postpartum ^(6)^.

Large-scale studies on postpartum BP variability are limited, and comprehensive data remain insufficient. Izumi et al. ^(7)^ examined daily self-measured BP in 370 pregnant Japanese women. Among normotensive women, BP began to increase from postpartum day 3, with sBP peaking at postpartum day 8 (+4.9 mmHg) and dBP peaking at postpartum day 7 (+4.7 mmHg) ^(7)^. sBP returned to predelivery levels by postpartum day 13, while dBP normalized by postpartum day 23 ^(7)^. In contrast, the postpartum BP in women with HDP gradually decreased after the first postpartum week. However, the degree of this decline varied depending on factors such as race and body mass index ^(8)^. A study conducted in the United States on 1,077 women with HDP investigated postpartum BP fluctuations and found that both sBP and dBP of women declined within the first 3 weeks postpartum and remained stable up to 6 weeks postpartum ^(9)^.

### Timing and frequency of BP measurement

#### During pregnancy

In Japan, it is recommended that BP be measured at every prenatal checkup, including the first visit during early pregnancy ^(10)^. The American College of Obstetricians and Gynecologists (ACOG) also includes routine BP measurements at every prenatal visit in its Guidelines for Perinatal Care ^(11)^. Similarly, the National Institute for Health and Care Excellence (NICE) in the United Kingdom emphasizes BP measurement at each prenatal checkup in its Antenatal Care Guidelines ^(12)^. However, evidence regarding the optimal frequency of BP measurement throughout pregnancy remains limited. A randomized controlled trial conducted in the United States and the United Kingdom compared pregnancy outcomes between women who received 13-14 prenatal visits and those who received 6-9 visits. The study found no significant differences in the incidence of HDP or related hospitalizations ^(13), (14)^. Nevertheless, no study has specifically evaluated the optimal frequency of BP measurement during pregnancy using the primary outcomes of early detection and treatment of HDP to improve perinatal outcomes, which is the fundamental purpose of BP monitoring in prenatal care.

#### Postpartum period

In Japan, postpartum health checkups are commonly conducted approximately 2 weeks and 1 month after delivery to assess the health of postpartum women. Although the guidelines for obstetric practice in Japan do not specifically mention BP measurement during postpartum checkups ^(10)^, BP is measured at almost all medical institutions in clinical practice. According to the Guidelines for Perinatal Care published by the ACOG, all postpartum women should undergo a postpartum checkup within 6 weeks of delivery, including BP measurement ^(11)^.

Compared with postpartum women without HDP, it is essential to monitor BP more carefully in postpartum women with HDP. The ACOG Guidelines for Perinatal Care recommend that women with severe HDP or those on antihypertensive medication undergo BP measurements approximately 72 hours after hospital discharge ^(11)^. For other women with HDP, a BP check is recommended 7-10 days postpartum ^(11)^. Similarly, the NICE guidelines recommend that postpartum women with preeclampsia who are on antihypertensive medication should have their BP monitored every 1-2 days until the medication is discontinued or BP normalizes ^(15)^. In Japan, postpartum women with HDP are typically instructed, prior to hospital discharge, to undergo home-based BP monitoring. If elevated BP was observed at home, patients were advised to visit the outpatient clinic. Even if BP is within the normal range at discharge, an initial postpartum checkup is usually scheduled within 1-2 weeks to evaluate BP. Thereafter, postpartum follow-up and BP monitoring are continued until approximately 12 weeks postpartum or until BP normalizes without the need for antihypertensive medication. However, there is no clear evidence regarding the optimal timing and frequency of postpartum BP measurement. Further research is required to establish guidelines for effective monitoring of postpartum BP.

### Significance and necessity of BP measurement

#### During pregnancy

Measuring BP during pregnancy allows for the assessment of HDP risk and prediction of its onset. Additionally, in cases where HDP develops, early diagnosis through BP monitoring facilitates the timely initiation of appropriate treatment, which is crucial for improving maternal and fetal outcomes.

Many women of reproductive age have few opportunities to undergo BP measurement, and for some, their first experience with regular BP monitoring occurs during prenatal checkups. This makes BP measurement during prenatal visits an effective tool for identifying women with undiagnosed essential or secondary hypertension. As hypertensive pregnancy disorders are major risk factors for superimposed preeclampsia, early diagnosis and appropriate management from early pregnancy are essential ^(16)^. Even among women without preexisting hypertension, attention to BP variability patterns from early pregnancy to 20 weeks of gestation has been reported to help predict and stratify the risk of developing HDP ^(17)^. Therefore, longitudinal BP monitoring during pregnancy is highly valuable for the risk assessment and early detection of HDP.

HDP often presents with few or no symptoms in its early stages, making BP measurement during prenatal checkups a crucial diagnostic tool. Many cases of HDP are first identified during routine BP checks at prenatal visits. This highlights that BP measurement is the simplest and most effective method for diagnosing HDP. Once HDP is diagnosed, its severity must be assessed immediately. In particular, an sBP ≥160 mmHg and/or a dBP ≥110 mmHg is classified as severe HDP ^(18)^, which is associated with an increased risk of serious maternal and fetal complications, including HELLP (Hemolysis, Elevated Liver enzymes and Low Platelets) syndrome, eclampsia, cerebral hemorrhage, and uteroplacental dysfunction. Given these risks, strict monitoring and intensive treatment are necessary, emphasizing the critical role of BP measurement. Additionally, cases of preeclampsia occurring before 34 weeks of gestation are associated with higher rates of perinatal complications ^(19)^, an increased risk of recurrent preeclampsia in future pregnancies ^(20)^, and a greater likelihood of long-term cardiovascular events in the mother compared with cases of preeclampsia developing later in pregnancy ^(21)^. Therefore, regular BP monitoring at each prenatal visit is essential to track BP trends and identify the timing of hypertension onset, which has significant implications for maternal and fetal health.

#### Postpartum period

Measuring BP during the postpartum period is essential for assessing the severity and progression of HDP and confirming its resolution. Generally, HDP is primarily managed through delivery, and BP often improves after childbirth. However, in some cases, BP may increase postpartum, particularly after hospital discharge, due to factors such as sleep deprivation associated with childcare and breastfeeding. In such situations, BP monitoring plays a critical role in early diagnosis of worsening hypertension and facilitates appropriate treatment. To confirm the resolution of HDP, BP must be monitored to ensure that it normalizes within 12 weeks postpartum. If hypertension persists beyond 12 weeks, essential or secondary hypertension should be considered. Additionally, although rare, de novo postpartum-onset HDP, which develops between 48 hours and 6 weeks after delivery, has been reported ^(22)^. Postpartum BP measurement is crucial for early detection and management of this condition.

In addition, long-term monitoring is necessary because approximately one-third of women with a history of HDP develop chronic hypertension within 10 years ^(23)^ and have an increased lifetime risk of cardiovascular disease ^(24)^, cerebrovascular events, and chronic kidney disease (CKD) ^(25)^. In Japan, workplace health examinations at least once a year are mandatory to provide regular BP assessments to working women. However, women who are not in the workforce, such as homemakers or those on maternity or childcare leave, are generally not eligible for these occupational health checkups and may not undergo routine BP measurements until they qualify for specific health checkups at the age of 40. This creates a significant gap in the early detection and management of postpartum hypertension in populations that are already at elevated risk. Establishing short- and long-term postpartum follow-up systems for high-risk women is an urgent public health concern. Recent randomized controlled trials have demonstrated that self-monitoring postpartum BP in women with HDP, combined with tailored antihypertensive treatment, leads to lower BP levels at six months ^(26)^, nine months ^(27)^, and three to four years postpartum ^(28)^. These findings underscore the importance of continuous postpartum BP monitoring and appropriate management of women with a history of HDP.

#### Significance of home BP monitoring during the perinatal period

In 2008, the AHA acknowledged that home BP monitoring was theoretically the most suitable method for monitoring BP changes during pregnancy, emphasizing its ability to allow multiple measurements at the same time each day over a prolonged period ^(29)^. Similarly, a report by the European Society of Hypertension noted that home BP monitoring during pregnancy could improve BP management in pregnant women ^(30)^. According to the Japanese Society of Hypertension guidelines, when there is a discrepancy between office and home BP monitoring readings, priority should be given to home BP monitoring results ^(1)^. In general, the more frequently the BP is measured, the closer the average BP value is to the true BP. Thus, it is recommended that home BP measurements should be performed twice per session, and the average of these two measurements should be used as the representative BP value for that session ^(1)^.

Home BP monitoring allows for the detection of white coat hypertension, a condition in which BP readings are elevated in clinical settings but remain normal outside of the clinic, typically at home. This enables a more comprehensive assessment of BP status. A meta-analysis of 12 studies using 24-hour ambulatory BP monitoring found that pregnant women diagnosed with white coat hypertension had a higher risk of developing preeclampsia compared to normotensive women (relative risk [RR] 2.29, 95% confidence interval [CI]: 1.18-4.43) ^(31)^. However, when compared to pregnant women with either HDP or chronic hypertension, their risk of developing preeclampsia was significantly lower (RR 0.39, 95% CI: 0.27-0.62) ^(31)^. These findings suggest that identifying white coat hypertension is important for risk stratification in pregnancy.

In recent years, research on home BP monitoring during pregnancy has expanded rapidly, highlighting its growing importance in maternal healthcare. In early pregnancy, home BP was more strongly correlated with neonatal birth weight than clinical BP measured during prenatal checkups ^(32)^. It was also found that not only the mean early pregnancy BP but also its trajectory over time was associated with birth weight ^(33)^. A systematic review of 18 studies (28,094 patients) demonstrated that remote BP monitoring in pregnant women at high risk for HDP reduced antenatal outpatient visits and hospital admissions without increasing adverse maternal and fetal outcomes or affecting psychosocial outcomes ^(34)^. Nevertheless, the BUMP1 trial, reported in 2022, investigated pregnant women at high risk of preeclampsia and found that BP self-monitoring via remote monitoring did not significantly improve the early detection of hypertension in outpatient settings compared with standard care ^(35)^. In addition, the BUMP2 trial examined pregnant women with chronic or gestational hypertension and found that remote BP self-monitoring did not significantly improve office BP control ^(36)^. A possible explanation for these findings is that the same diagnostic criteria were applied for both office and home BP measurements, which may have influenced the results. However, a recent meta-analysis suggested that BP telemonitoring significantly reduced the need for labor induction and was associated with favorable neonatal birth weight outcomes ^(37)^. Other perinatal outcomes were comparable to those of office BP monitoring, with no increase in the risk of adverse events ^(37)^.

### Management of abnormal findings

**Figure 3.**
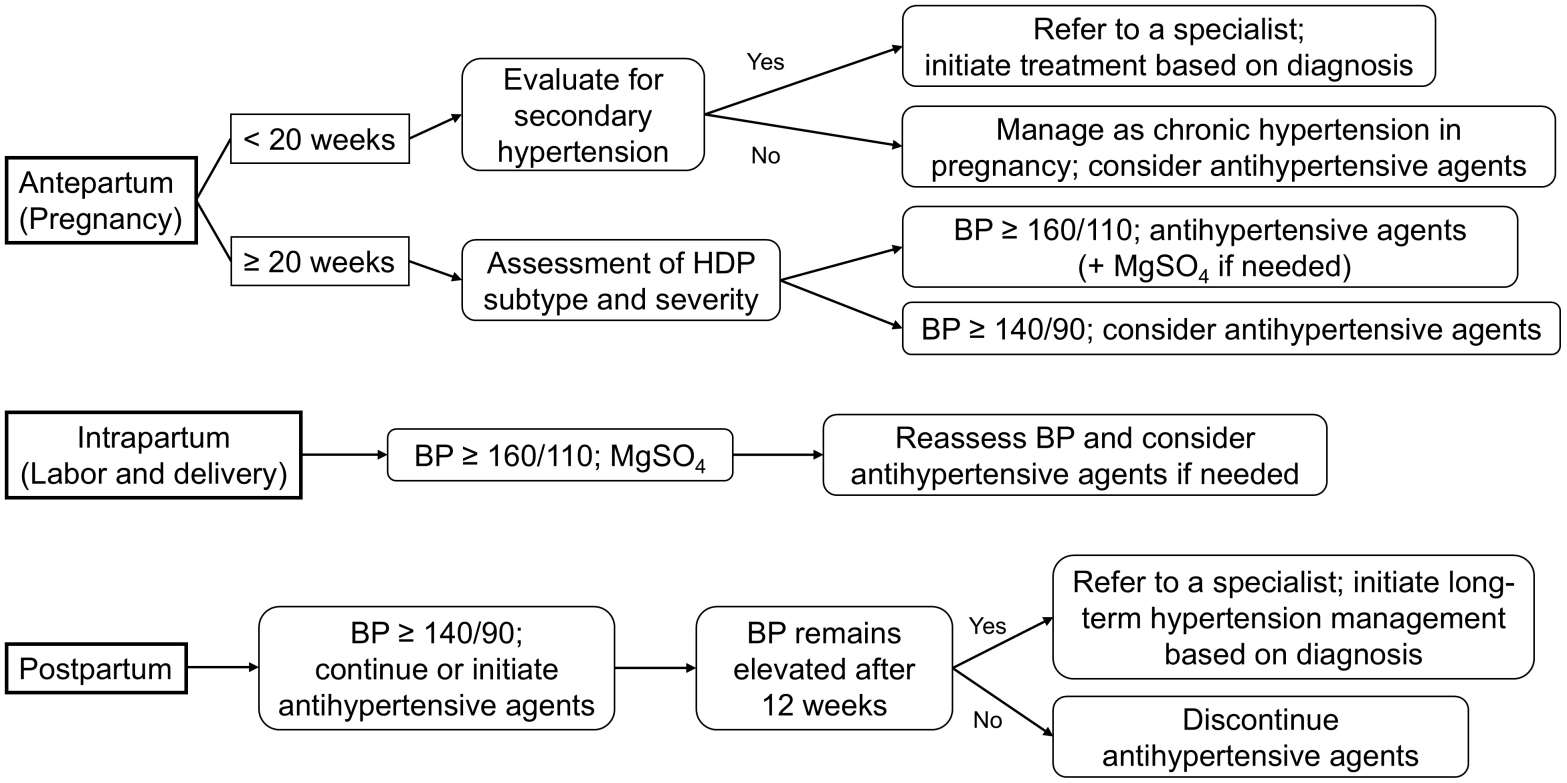
Clinical algorithm for hypertension management across the perinatal period. BP: blood pressure; HDP: hypertensive disorders of pregnancy; MgSO_4_: magnesium sulfate.

An overview of the clinical management of abnormal BP during pregnancy, labor, and the postpartum period is illustrated in Figure 3.

#### During pregnancy

If hypertension is detected before 20 weeks of gestation, including at the initial prenatal visit during early pregnancy, it should be presumed to have occurred before conception. In such cases, the condition is classified as chronic hypertension during pregnancy, a subtype of HDP ^(18)^. In pregnancies complicated by chronic hypertension, it is crucial to differentiate between essential and secondary hypertension. Early consultation with specialist departments such as the cardiology or endocrinology departments is recommended to facilitate differential diagnosis through blood tests and imaging studies. Once diagnosed, appropriate medical or surgical treatment should be initiated promptly. It is well established that pregnancies complicated by secondary hypertension have worse perinatal outcomes for both the mother and fetus compared to those complicated by essential hypertension ^(38)^. Therefore, such cases require management at a high-level perinatal care center to ensure optimal maternal and fetal outcomes.

If hypertension is detected after 20 weeks of gestation, further evaluation should be conducted using urinalysis and blood tests to assess the presence of maternal organ dysfunction. Simultaneously, ultrasound examinations and fetal heart rate monitoring should be performed to evaluate the fetoplacental function. Based on these findings, appropriate classification of the HDP subtype and severity assessments should be carried out. Since HDP is a progressive condition, patients diagnosed with preeclampsia or superimposed preeclampsia require hospitalization, with BP measurements conducted three to four times daily and blood tests performed at least twice per week ^(10)^. There is insufficient evidence regarding the need for hospitalization in HDP without severe features. However, twice-weekly clinic visits are recommended if the patients are managed on an outpatient basis. Additionally, home BP monitoring should be encouraged to facilitate early detection of disease progression. If the BP shows an increasing trend or severe hypertension develops, immediate hospitalization is required for intensive monitoring and management.

Regardless of the HDP subtype, antihypertensive therapy should be initiated when sBP reaches ≥160 mmHg and/or dBP reaches ≥110 mmHg ^(3), (10)^. Additionally, in cases of severe hypertension, magnesium sulfate should be administered as seizure prophylaxis ^(10), (39)^. In the specific context of severe hypertension during labor and delivery, seizure prophylaxis with magnesium sulfate is often prioritized as the initial intervention, as it may sufficiently reduce BP without the immediate need for antihypertensive agents ^(10)^. The necessity of antihypertensive treatment should therefore be reassessed after magnesium administration. There remains, however, no evidence-based BP target for antihypertensive treatment, which makes this an important subject for future research. There is no universal consensus regarding the necessity of antihypertensive treatment for nonsevere hypertension. However, the International Society for the Study of Hypertension in Pregnancy and the NICE recommend initiating antihypertensive therapy when BP exceeds 140/90 mmHg ^(15), (39)^, with NICE setting the target BP at ≤135/85 mmHg ^(15)^. A systematic review reported that in pregnant women with nonsevere hypertension, antihypertensive treatment reduced the incidence of severe hypertension compared with no treatment or placebo ^(40)^. However, the treatment did not affect the incidence of severe preeclampsia, fetal or neonatal mortality, or intrauterine growth restriction ^(40)^. In contrast, in pregnancies complicated by chronic hypertension, lowering the sBP to <130 mmHg during early pregnancy (16-19 weeks) has been associated with a reduced risk of developing superimposed preeclampsia ^(41)^.

#### Postpartum period

At the postpartum checkup (2 weeks or 1 month postpartum), if hypertension is detected, antihypertensive therapy should be initiated based on severity. If antihypertensive treatment was initiated before delivery, it should be continued postpartum. Whenever possible, home BP monitoring should be encouraged, and regular outpatient visits with BP measurements should be maintained until BP normalizes without the need for antihypertensive therapy. If hypertension persists beyond 12 weeks postpartum, it is presumed that chronic hypertension existed before pregnancy, and the patient should be referred promptly to a specialist. Additionally, even before 12 weeks postpartum, early referral to a specialist should be considered in cases of treatment-resistant or persistent hypertension with no signs of improvement.

## Urinary Protein

### Overview of examination

The urine protein test primarily detects and evaluates albumin in urine. Urine protein levels graded 1+, 2+, and 3+ corresponded to approximately 30-100, 100-300, and 300-1,000 mg/dL, respectively. Proteinuria can be observed in conditions such as acute and chronic nephritis, pyelonephritis, nephrotic syndrome, fever, fatigue, and renal damage associated with systemic diseases. Recently, test strips that semiquantitatively measure urinary creatinine and express the results as protein-to-creatinine ratios (PCRs) have become available.

Physiological changes in the urinary system during pregnancy can influence the urinalysis results. Renal plasma flow increases by up to 80% in early pregnancy and decreases in late pregnancy ^(42)^. The glomerular filtration rate (GFR) increases by approximately 50% throughout pregnancy ^(42)^. Generally, urinary glucose levels increase owing to enhanced GFR and reduced proximal tubular reabsorption ^(43)^. Similarly, urinary protein levels are higher during pregnancy than in the nonpregnant state, with spot urine PCR increasing as the pregnancy progresses ^(44)^. The reported ranges are 0.011-0.027 in early pregnancy, 0.024-0.048 in mid-pregnancy, and 0.043-0.150 in late pregnancy ^(44)^. These elevated renal plasma flow and GFR levels return to pre-pregnancy values within 6-8 weeks postpartum ^(45)^.

### Timing and frequency of examination

In Japan, obstetrical practice guidelines recommend performing urinalysis, including semiquantitative tests for urine glucose and urine protein, at every prenatal checkup ^(10)^. Similarly, in the United States, the Guidelines for Perinatal Care published by the ACOG make the same recommendations ^(11)^. In contrast, the Antenatal Care Guidelines issued by NICE in the United Kingdom suggest testing only for urine proteins ^(12)^. Regarding postpartum examinations, it is common practice in Japan to record the results of qualitative urine protein tests in the Maternal and Child Health Handbook during postpartum checkups. These checkups are typically conducted around 1 month after delivery, but in recent years, examinations around 2 weeks postpartum have also become increasingly common. However, neither the Japanese guidelines for obstetric practice nor the ACOG guidelines specifically mention postpartum urinalysis. Similarly, the NICE guidelines do not address postpartum urinalysis, except in cases of preeclampsia, where qualitative urine protein testing is recommended 6-8 weeks postpartum ^(12)^.

### Significance and necessity of examination

Urinary protein testing during pregnancy may play a vital role in the early diagnosis and prediction of preeclampsia. Proteinuria has historically been considered a symptom of toxemia of pregnancy and was once considered sufficient for its diagnosis. However, with evolving concepts and definitions of HDP, proteinuria is now recognized as one of the diagnostic criteria for preeclampsia ^(18)^. Proteinuria that occurs after 20 weeks of gestation and resolves postpartum is referred to as gestational proteinuria ^(18)^. Although gestational proteinuria can occur without concurrent hypertension, a significant proportion of cases progress to preeclampsia. Nearly 30% of gestational proteinuria cases progress to preeclampsia ^(46), (47)^. Among preeclampsia cases with proteinuria, 25% were preceded by proteinuria and 75% by hypertension ^(48)^. Moreover, preeclampsia preceded by gestational proteinuria was associated with significantly earlier delivery (mean gestational age 32.5 weeks vs. 36.1 weeks, p < 0.001) compared with preeclampsia not preceded by proteinuria ^(49)^. In addition, studies have shown that among women who developed eclampsia, a substantial proportion had preexisting preeclampsia (17.8%-57.2%) or gestational hypertension (9.3%-21.8%) during prior checkups ^(50)^. Notably, 7.5%-9.3% of these women had isolated gestational proteinuria without hypertension ^(50)^. However, no large-scale studies have evaluated the significance of urine protein testing as a screening tool for preeclampsia in asymptomatic pregnant women ^(51)^. A prospective study from Australia―where proteinuria is defined as 1+ or higher, unlike Japan―found no significant difference in the subsequent development of preeclampsia between women with and without proteinuria at their initial visit ^(52)^. The study concluded that routine proteinuria testing did not contribute to the early detection of preeclampsia ^(52)^. Proteinuria during pregnancy remains an important marker that may indicate the future onset of preeclampsia or eclampsia and should not be overlooked. However, it is important to note that high-quality evidence supporting the efficacy of proteinuria as a predictive tool for the early diagnosis of preeclampsia or eclampsia is lacking. Furthermore, the optimal frequency of urinary protein testing during pregnancy has not yet been established. This highlights the need for further research to clarify the role of urinary protein testing in predicting HDP and to determine the best practices for clinical application.

The primary purpose of urinary protein testing in the postpartum period is to confirm the resolution of proteinuria associated with preeclampsia and to screen for other underlying renal diseases. By definition, proteinuria associated with preeclampsia should resolve by 12 weeks postpartum ^(18)^. On average, hypertension, proteinuria, and renal dysfunction in patients with preeclampsia improve within 35.8 days after delivery ^(53)^. However, women with preeclampsia and proteinuria are at an increased risk of developing CKD ^(54)^. Therefore, proteinuria identified during the postpartum checkup, typically conducted approximately 1 month after delivery, requires continued follow-up. If it persists beyond 12 weeks postpartum, referral to a specialist and further evaluation―potentially including renal biopsy―is warranted. In a study of 34 patients with preeclampsia and persistent proteinuria beyond 12 weeks postpartum, renal biopsy was performed in 14 patients, revealing underlying kidney diseases such as membranoproliferative glomerulonephritis and IgA nephropathy in 10 patients ^(55)^.

In the diagnostic criteria for HDP, a PCR ≥0.3 g/gCr is generally considered positive for proteinuria ^(18)^. However, the clinical practice guideline for CKD in Japan defines proteinuria as a PCR ≥0.15 g/gCr ^(56)^, considers it a risk factor for future CKD, and recommends long-term monitoring. This discrepancy implies that women with a PCR of 0.15-0.3 g/gCr, classified as negative for proteinuria in obstetric care, may lose critical follow-up for CKD risk. Therefore, the establishment of unified criteria for postpartum proteinuria is urgently required. Conversely, the utility of qualitative urinary protein testing in postpartum women without prior gestational proteinuria or preeclampsia remains uncertain and has not been established. Further research is required to clarify its significance in these populations.

### Management of abnormal findings

For urinary protein testing, semiquantitative screening results are considered positive if 1+ or higher proteinuria is detected on two or more consecutive occasions, or if a single result of 2+ or higher is observed ^(10)^. A positive urinary protein screening result is further confirmed as proteinuria if the 24-hour urinary protein excretion is ≥300 mg or the PCR in a spot urine sample is ≥0.3 g/gCr. Notably, semiquantitative urine protein tests are prone to false positives, with positive predictive values of 22% for 1+, 79% for 2+, and 99% for 3+ ^(57)^. If neither a 24-hour urinary protein test nor a spot urine PCR test can be performed, a diagnosis of proteinuria may be made solely on the basis of positive semiquantitative urinary protein screening results.

There is insufficient evidence regarding the optimal management of pregnant women with isolated proteinuria, and the most appropriate approach remains unclear. If persistent proteinuria is observed before 20 weeks of gestation, referral to a nephrologist may be advisable to assess underlying renal disorders. In contrast, if proteinuria develops after 20 weeks, continued follow-up is warranted, considering the potential onset of preeclampsia. Measurement of the soluble fms-like tyrosine kinase-1:placental growth factor (sFlt-1:PlGF) ratio may also assist in differentiating preeclampsia from underlying renal disease. In a multinational study, an sFlt-1:PlGF ratio ≤38 was shown to have a negative predictive value of 99.3% for the development of preeclampsia within 1 week among women with suspected disease ^(58)^. Although diagnostic cut-off values have not been specifically validated for isolated proteinuria, they may aid in clinical decision-making in ambiguous cases. One study reported a median sFlt-1:PlGF ratio of 436 in women with preeclampsia and 4.0 in those with CKD, suggesting a marked difference in angiogenic profiles ^(59)^.

At the 1-month postpartum checkup, if qualitative testing for proteinuria yields a result of ± or higher, the PCR in a spot urine sample should be measured. As mentioned earlier, in obstetric practice, a PCR ≥0.3 g/gCr is generally considered positive for proteinuria. However, taking into account the thresholds set forth in the CKD guidelines, it is advisable to consider a PCR ≥0.15 g/gCr as indicative of proteinuria. In such cases, follow-up should be continued until 12 weeks postpartum to confirm resolution of proteinuria. If proteinuria persists beyond 12 weeks postpartum, referral to a nephrologist is recommended. This approach aligns with the guidelines issued in the United Kingdom ^(15)^, which similarly advocate specialist referral in such cases. Furthermore, even before 12 weeks postpartum, early referral to a nephrologist should be considered if severe proteinuria persists, and preeclampsia has been ruled out as the underlying pathology.

## Edema

### Overview of examination

Edema is defined as swelling in a specific region due to excessive accumulation of interstitial fluid within soft tissues outside the capillaries ^(60), (61), (62)^. Clinical assessment involves evaluation of its distribution and characteristics. Edema can be categorized, based on distribution, into localized and systemic edema ^(60), (62)^. Localized edema manifests asymmetrically, whereas systemic edema presents symmetrically, predominantly affecting areas prone to lower tissue pressure or increased hydrostatic pressure, such as the eyelids, lower legs, and foot dorsum. Characteristic evaluation primarily involves palpation and application of firm pressure on bony regions, such as the anterior tibia or ankles, for approximately 10 seconds to determine the presence of pitting. Pitting edema, characterized by a depression after pressure application, commonly occurs in pathological states such as nephrotic syndrome, liver cirrhosis, and heart failure. Most cases of edema are classified as pitting, while non-pitting edema occurs in limited conditions such as lymphedema or myxedema, making it relatively rare.

Conditions that cause edema include nephrotic syndrome, liver cirrhosis, congestive heart failure, and protein-energy malnutrition ^(60), (62), (63)^. However, most cases of edema in pregnancy are attributed to physiological changes ^(64)^. During pregnancy, sodium is retained because of the influence of estrogen and activation of the renin-angiotensin system. Approximately 6.5 L of water is retained throughout pregnancy, with the plasma volume increasing from 1,200 to 1,300 mL ^(65)^. Consequently, serum sodium levels decrease, reflecting reduced maternal plasma osmolality ^(65)^. Additionally, an enlarged uterus compresses the inferior vena cava, impairing venous return from the lower extremities. These factors collectively result in edema, predominantly in the lower limbs, during the later stages of pregnancy ^(66)^. Nevertheless, it should also be noted that some cases of edema in pregnancy are caused by serious conditions associated with severe outcomes, such as HDP, deep vein thrombosis (DVT), and peripartum cardiomyopathy.

Excessive maternal weight gain may serve as an indirect indicator of edema and should be carefully monitored. A study by Morikawa et al. ^(67)^ reported that in women with preeclampsia, gestational weight gain exceeding 1.5 kg during the week prior to delivery was significantly associated with life-threatening maternal complications. Their findings highlight the clinical importance of tracking short-term weight changes in late pregnancy as a potential predictor of maternal risk.

### Timing and frequency of examination

Since edema can be assessed through visual inspection and palpation at no cost, assessments can be conducted for all pregnant women during routine antenatal checkups. However, the significance and necessity of such examinations lack clear evidence.

### Significance and necessity of examination

Edema detection during antenatal checkups is valuable for diagnosing and managing conditions such as HDP and DVT. Guidelines for obstetric practice in Japan explicitly include the “evaluation of edema” as a routine examination ^(10)^. In contrast, guidelines published by ACOG ^(11)^ and NICE ^(12)^ do not specifically mention edema evaluation during routine checkups. Nonetheless, the antenatal care forms exemplified in the ACOG guidelines include a section documenting edema, suggesting that its identification is considered clinically significant ^(11)^.

Currently, no study has conclusively demonstrated that edema assessment during antenatal checkups improves pregnancy and delivery outcomes ^(68)^. Therefore, routine practice requires further validation. Nonetheless, because the examination is simple, cost-effective, and imposes minimal burden on pregnant women, there is no compelling rationale for omitting it.

### Management of abnormal findings

When edema is observed during pregnancy, it is crucial to investigate underlying diseases or pathological conditions. However, because edema, particularly in the lower limbs, frequently occurs as a physiological change associated with pregnancy, it is essential to comprehensively evaluate not only the presence of edema but also the results from other assessments, such as BP, urine protein, and blood tests, to identify the underlying cause ^(60), (62), (63)^. Key factors to assess include edema distribution, i.e., localized or systemic; type of edema, i.e., pitting or non-pitting; and the presence of redness, tenderness, or warmth. Physiological edema during pregnancy is often localized (sometimes systemic), pitting, and resolves slowly.

If unilateral lower-extremity swelling accompanied by redness, pain, and warmth occurs suddenly or over a few days, DVT should be suspected, warranting additional tests such as lower-extremity venous ultrasonography and D-dimer assays, along with consultation with relevant specialists. Venous thromboembolism, which encompasses both DVT and pulmonary embolism, is five times more likely to occur during pregnancy compared with the nonpregnant state and represents a leading cause of maternal mortality ^(69), (70)^. The risk of venous thromboembolism persists throughout all trimesters of pregnancy, with the highest risk typically observed in the third trimester ^(64)^. Therefore, when a pregnant woman presents with leg edema, DVT should be considered the primary differential diagnosis and subsequently excluded.

In cases where edema is accompanied by elevated BP and/or proteinuria, HDP should be highly suspected, and as outlined in the section on BP in this paper, appropriate management should be followed. Systemic edema with dyspnea or shortness of breath may indicate peripartum cardiomyopathy and should be evaluated via electrocardiography, chest radiography, and B-type natriuretic peptide blood testing, with referral to specialized care. Peripartum cardiomyopathy is an extremely rare condition in Japan, with an incidence of approximately one case per 20,000 deliveries ^(71)^. However, because it is a life-threatening disease for the mother, it is essential to reliably differentiate peripartum cardiomyopathy in pregnant women presenting with edema.

If DVT, HDP, and peripartum cardiomyopathy are excluded, further tests such as blood analysis and urinalysis should be conducted to investigate other potential causes, including nephrotic syndrome, renal failure, hepatic failure, nutritional deficiencies, and vasculitis. When no underlying cause is identified, edema should be considered a physiological change associated with pregnancy.

## Conclusions

The triad of BP measurement, urinalysis, and edema assessment plays a pivotal role in the prediction and diagnosis of HDP. These examinations have contributed not only to HDP management but also to the identification and diagnosis of various other pregnancy complications.

BP measurement is the most robustly validated component of this triad, with substantial evidence confirming its critical role in the detection and management of HDP. Its systematic implementation in prenatal care has improved maternal and perinatal outcomes through timely intervention. Nevertheless, there is an urgent need to establish optimal BP thresholds and treatment targets during pregnancy. Home BP monitoring offers a promising avenue for addressing this knowledge gap by providing longitudinal data that may better reflect the true BP variability and potentially enhance the already proven clinical utility of BP assessments. One critical issue regarding urinary protein assessment requiring resolution is the establishment of standardized criteria for the follow-up of postpartum proteinuria. The discrepancy between obstetric and nephrological thresholds for defining significant proteinuria creates a gap in the continuity of care, highlighting the need for interdisciplinary consensus on appropriate monitoring protocols for women with persistent postpartum proteinuria. Edema evaluation is perhaps the most challenging factor of the triad, with limited evidence supporting its routine assessment. Nevertheless, because edema can occasionally manifest as a sign of a serious condition, maintaining its assessment in standard prenatal care appears prudent. While their relative clinical importance may vary depending on the context, it is important to interpret these three assessments collectively rather than in isolation. Recent advances in biomarker research, particularly involving the sFlt-1/PlGF ratio, offer promising tools for preeclampsia prediction and risk stratification. Future investigations should examine how these approaches can complement or potentially refine conventional methods such as BP, proteinuria, and edema assessments.

A notable characteristic of these three examinations is their noninvasive nature and minimal cost, making them accessible across diverse healthcare settings. If future research can establish their clinical utility through robust evidence, these examinations have the potential to contribute to both cost-effective healthcare and improved maternal and fetal outcomes. It is worth noting that in Japan, approximately half of pregnant women complete their entire perinatal care, from prenatal checkups to delivery and postpartum examinations, at primary care facilities, such as private clinics. Therefore, these simple examinations of BP, urinalysis, and edema assessment may remain particularly valuable in the Japanese healthcare context, provided that practitioners are well informed about both their utility and limitations. Rather than abandoning these traditional assessments, clarifying their precise role in contemporary obstetric practice through targeted research remains essential, optimizing their implementation to balance clinical benefits with resource utilization.

## Article Information

### Acknowledgments

We are profoundly grateful to the late Dr. Daisaku Maehara for his generous contribution of photographs and historical insights regarding the Maternal and Child Health Handbook. His invaluable knowledge has greatly enriched this review.

We would like to thank Editage (www.editage.com) for English language editing.

### Author Contributions

All authors contributed to the conception and design of the study. Yoshitsugu Chigusa, Kazuki Yamano, Taito Miyamoto, and Hirohito Metoki conducted the literature search, reviewed relevant studies, and wrote the first draft of the manuscript. Haruta Mogami, Masaki Mandai, and Akihiko Sekizawa made important contributions to the manuscript revision. Akihiko Sekizawa secured funding and supervised the entire study.

### Conflicts of Interest

None

### Approval by the Institutional Review Board

Ethical approval was not required for this narrative review as it did not involve primary research with human participants or animals.
